# Identification and classification of epithelial cells in nephron segments by actin cytoskeleton patterns

**DOI:** 10.1111/febs.15088

**Published:** 2019-11-01

**Authors:** Girishkumar Kaitholil Kumaran, Israel Hanukoglu

**Affiliations:** ^1^ Laboratory of Cell Biology Ariel University Ariel Israel

**Keywords:** aquaporin, cytokeratin, kidney, microvilli, papilla

## Abstract

The basic functional unit in a kidney is the nephron, which is a long and morphologically segmented tubule. The nephron begins with a cluster of capillaries called glomerulus through which the blood is filtered into the Bowman's space. The filtrate flows through the nephron segments. During this flow, electrolytes and solutes are reabsorbed by channels and transport systems into the capillaries wrapped around the nephron. Many questions related to renal function focus on identifying the sites of expression of these systems. In this study, we mapped whole kidney sections by confocal microscopic imaging of fluorescent phalloidin, which binds to actin filaments. In tile scans (composed of hundreds of images) of these sections, the cortex and the medullary regions (outer and inner stripes of the outer medulla, and inner medulla) could be easily identified by their cytoskeletal patterns. At a higher resolution, we identified distinct features of the actin cytoskeleton in the apical, basal, and lateral borders of the cells. These features could be used to identify segments of a nephron (the proximal tubule, thin and thick segments of Henle's loop, and distal tubule), the collecting duct system, the papillary ducts in the papilla, and the urothelium that covers the pelvis. To verify our findings, we used additional markers, including aquaporin isoforms, cytokeratin 8‐18, and WGA lectin. This study highlights the power of high‐resolution confocal microscopy for identifying specific cell types using the simple probe of F‐actin‐binding phalloidin.

## Graphical abstract

In this study, we mapped whole kidney sections by confocal microscopic imaging of fluorescent phalloidin that binds to actin filaments. At a high resolution, we identified actin cytoskeleton features of the apical, basal, and lateral borders of the epithelial cells to identify and distinguish between segments of a nephron (the proximal tubule, thin and thick segments of Henle's loop, and distal tubule) and the renal collecting duct system.

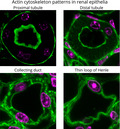

**Abbreviations**
AKI: acute kidney injury
AQP: aquaporin
CD: collecting duct
DCT: distal convoluted tubule
DT: distal tubule
IC: intracytoplasmic
JGA: juxtaglomerular apparatus
PT: proximal tubule

## Introduction

**Figure 1:**
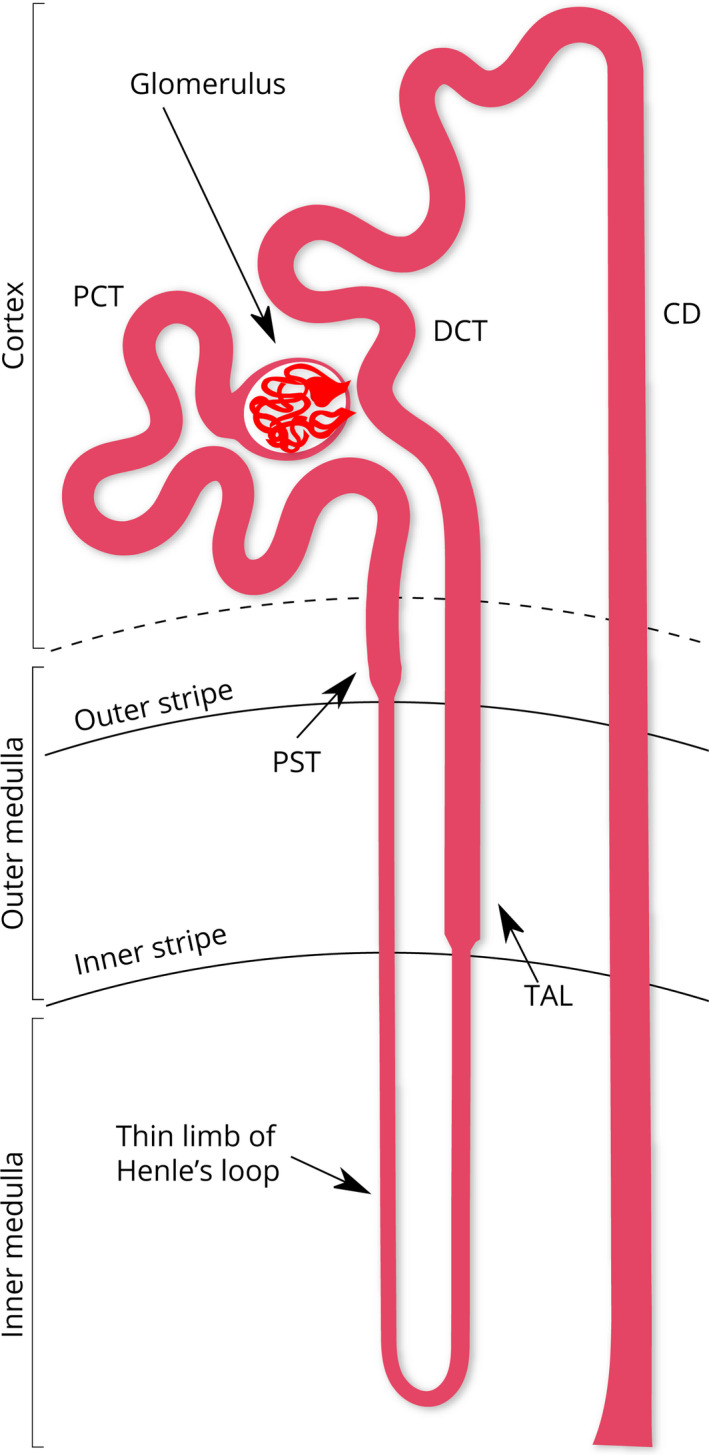
A diagram of the nephron and the localization of its segments in the cortex and the medullary regions. CD, collecting duct; DCT, distal convoluted tubule; PCT, proximal convoluted tubule; PST, proximal straight tubule and TAL, thick ascending limb. This figure is based on the nomenclature of kidney structures 3.

The kidney is a complex organ that filters the blood plasma continuously to maintain water and electrolyte homeostasis and acid–base balance. In humans, about a liter of blood flows through the kidney every minute 1. The basic functional unit in the kidney is the nephron, which is a long and morphologically segmented tubule 2, 3. In the human kidney, the average number of nephrons has been estimated to be in the range of a million, and in the mouse kidney about 20 000 4, 5, 6. The nephron begins with the Bowman's capsule, which circumscribes a cluster of capillaries called glomerulus (Fig. 1). The glomerular filtrate flows through the proximal tubule (PT), the loop of Henle, and the distal tubule (DT) segments of the nephron 7. At the end of the nephron, the collecting ducts (CD) merge into the papillary ducts that form the papilla. The mouse kidney is unilobar with a single papilla. The human kidney is multilobar with a range of 4–18 papillae^4^.

Most of the electrolytes and solutes in the filtrate are reabsorbed into the peritubular capillaries wrapped around the nephron. The reabsorption of the solutes and ions is dependent on the function of a wide variety of channels and transporters localized on the luminal membrane of the epithelial cells that form the nephron tubules 8. The coordinated action of the channels and transport systems in the nephron results in the fine‐tuning of the plasma composition that is essential for the normal functioning of the body 9. The expression and function of these systems are regulated by a complex array of hormones, including the renin–angiotensin–aldosterone system and vasopressin 10, 11, 12, 13. Many questions about the kidney function concentrate on understanding the sites of expression and the mechanism of regulation of the renal transport systems 14, 15, 16, 17. Methods that have been used to identify the segments of the nephron where each channel and transporter are located include fluctuation analysis, patch‐clamp techniques, immunohistochemistry, and western blots (WB) 18, 19, 20, 21, 22.

Our laboratory has been working on mapping the sites of localization of epithelial sodium channels and cystic fibrosis transmembrane conductance regulator in various epithelial tissues 23, 24, 25, 26, 27. We wanted to use similar techniques to map the localization of these channels in kidney sections but quickly faced the problem of the morphological complexity of the nephron segments and their identification. In our studies on the skin, the respiratory tract, and the male and female reproductive tract epithelia, we visualized cell borders using fluorescent phalloidin that binds specifically to actin filaments 28 to verify the structural integrity of tissue sections 24, 26. We hypothesized that by using a similar approach, we might be able to distinguish between different nephron segments.

In eukaryotic cells, there are three major types of cytoskeletal structures, microfilaments, intermediate filaments, and microtubules 29. In epithelial cells, intermediate filaments form a dense cytoplasmic network that is connected to the sites of cell–cell adhesion points. Microtubules are also located within the cytoplasm and are involved in the movement of vesicles and organelles. In contrast to these, microfilaments composed of actin filaments lie underneath the membrane providing structural support 30, 31, 32. Actin filaments form the backbone of microvilli that cover the surface of PTs, secondary foot processes in glomerular podocytes, and stereocilia that are found in specific epithelial cell types 27, 33, 34. Actin is a most highly conserved protein with six isoforms in mammals 35, 36. Four isoforms are expressed mainly in muscle cells, and two (β‐actin and γ‐cyto‐actin) are expressed ubiquitously as abundant cytoplasmic proteins that form the actin cytoskeleton 30, 36.

A first step in the diagnosis of renal diseases is the measurement of specific metabolites in serum, such as creatinine, the levels of which may be affected by the malfunction of the kidney tubules 37. Alterations in the levels of such biomarkers generally reflect structural damage to kidney tubules. For example, an early manifestation of acute kidney injury (AKI) is the disruption of the actin cytoskeleton, especially the microvilli in the PT 38, 39.

In the present study, we report that different tubular segments of the kidney can be identified by the simple patterns of the actin cytoskeleton using a fluorescent marker. We verified the criteria we established by additional markers, including aquaporin (AQP) isoforms, cytokeratin 8‐18, CD34 and WGA lectin. Overall, our findings allowed us to construct an identification table for various nephron segments based on the actin cytoskeleton features.

## Results

**Figure 2:**
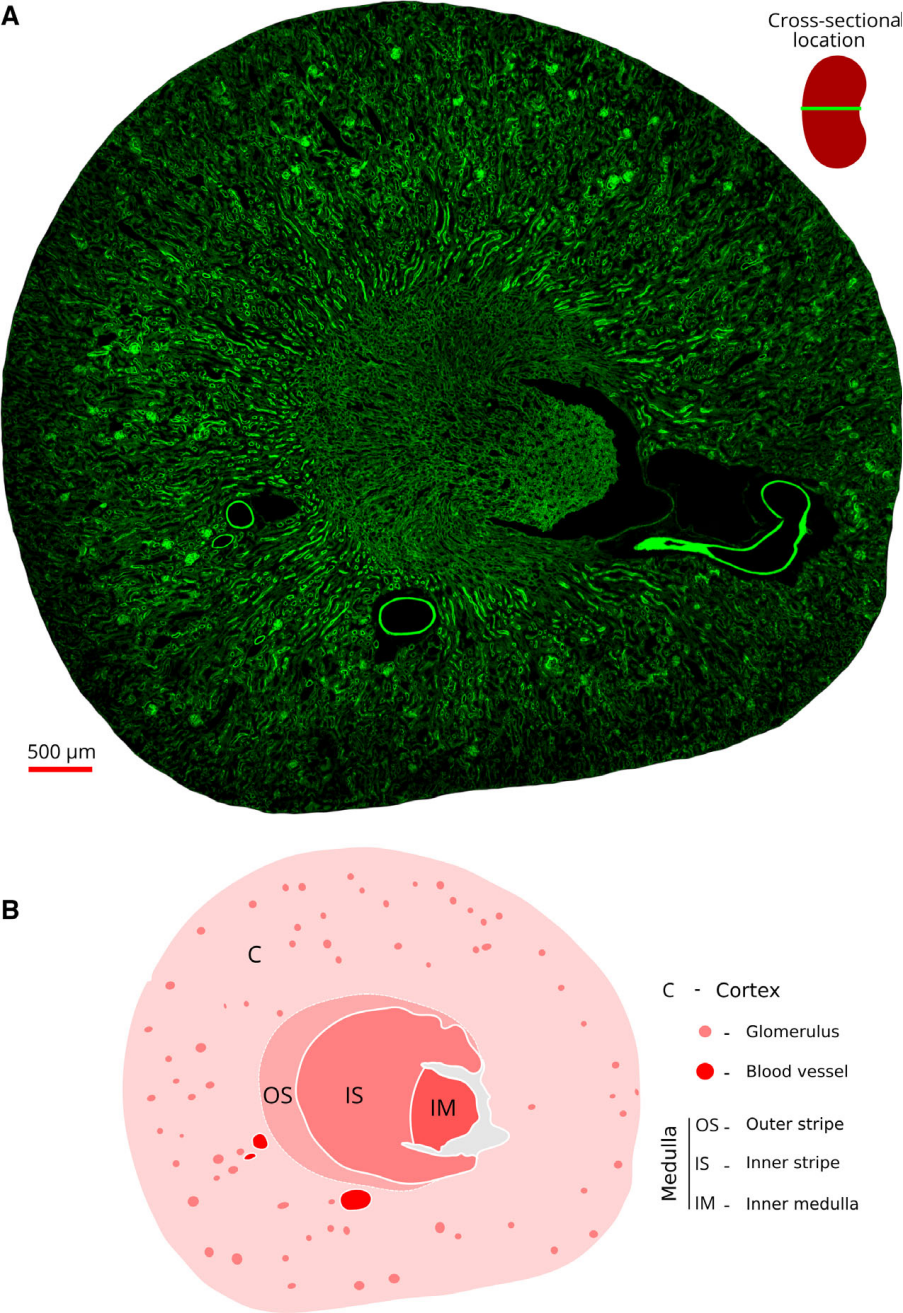
A whole mouse kidney section as visualized by actin filament fluorescence. (A) This cross‐sectional image was constructed by stitching 900 (30 × 30) partially overlapping confocal microscopy images taken in a single session of about 4 h. The kidney section (15 μm thick) was reacted with fluorescent phalloidin as described in Materials and methods section. The top right image shows the location of the section relative to the whole mouse kidney. The glomeruli can be easily identified as intensely stained small light‐green beads in the cortex. The transitional region between the cortex and the medulla is characterized by intensely stained long stretches of tubules around the region of the arcuate blood vessels. (B) A schematic map of the section marking the kidney regions that can be distinguished by the actin staining patterns shown in A. The regions marked in the cortex include the glomeruli, and the regions in the medulla include the outer and inner stripes and the inner medulla (see the legend on the right side).

Initially, we generated whole‐section images of mouse kidney by staining with fluorescent‐labeled phalloidin. The cross‐sectional images from different parts of the kidney showed clear regional differences in the distribution of the actin cytoskeleton. In Fig. 2, we present a cross‐sectional image from about the middle of a mouse kidney to show the differences between the cortex and the medullary regions. The schematic map in Fig. 2B presents a key for the identification of the locations of the cortex, inner and outer stripe of the outer medulla, and inner medulla based on their distinct actin cytoskeleton patterns.

In the sections below, we present the distinct characteristics of the actin cytoskeleton for each region of the kidney.

### Cortex

In mammals, the nephron has two parts: the renal corpuscle and the renal tubule. The renal corpuscle consists of the glomerulus and the Bowman's capsule. In the cortex, the glomeruli could be identified easily as beads of intensely stained actin filaments (Fig. 2). Note that the medulla is devoid of glomeruli.

**Figure 3:**
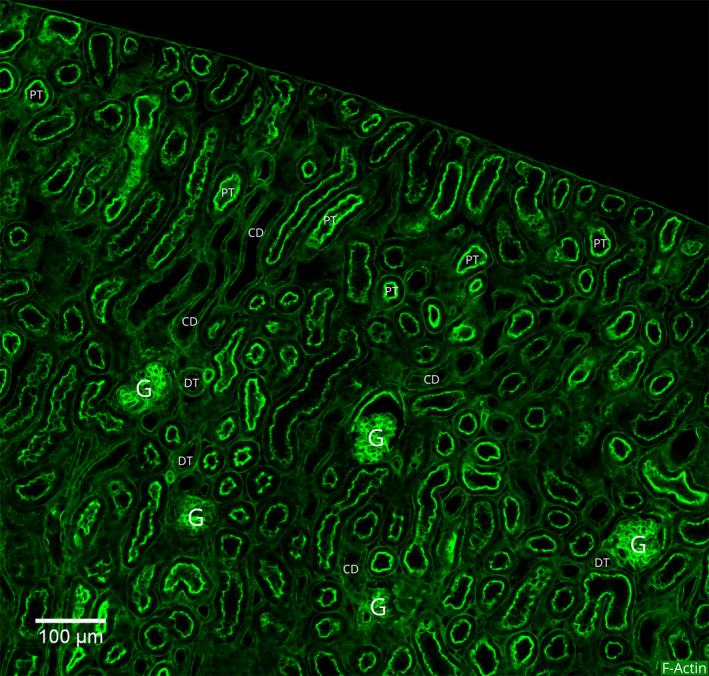
A magnified view of the actin cytoskeleton patterns in the mouse kidney cortex. This image of a portion of the kidney cortex was constructed by tile‐scanning 25 (5 × 5) confocal microscopy images. Five glomeruli appear as intensely stained beads of actin filaments in two rows (three in the top row and two in the bottom row). A majority of the tubule segments seen in this area have thick apical and thin basal actin filament bundles. There are also some tubules with a thin lining of actin filament bundles on both apical and basal membranes. Markings: G, glomerulus; PT, proximal tubule; DT, distal tubule; and CD, collecting duct.

At higher magnification, we identified three distinct types of tubular epithelia based on the actin fluorescence (F) (Fig. 3):

- A thick wavy line at the apical border and a thin line on the basal border,
- A thin line on both the apical and basal borders, and
- A thin line on the apical, basal, and lateral borders.

As described below, we could associate each one of these distinct cytoskeletal profiles with a specific renal tubule segment (Table 1).

**Table 1:** Actin cytoskeleton patterns and morphometric parameters of the epithelial cells of the nephron segments, papillary ducts, and the urothelium.

Epithelia	Region	Markers	Cell height (µm)^a^	Actin cytoskeleton				
				Microvilli	Apical	Basal	Lateral	IC
Proximal tubule	Cortex	AQP‐1	9.8 ± 1.7	**+**	**+**	**+**		
Distal convoluted tubule	Cortex	WGA Lectin	6.7 ± 1.9		**+**	**+**		**+**
Collecting duct	Cortex	AQP‐2/CK8‐18	5.9 ± 1.9		**+**	**+**	**+**	
	Outer medulla	AQP‐2/CK8‐18	4.1 ± 1.2					
	Inner medulla	AQP‐2/CK8‐18	3.4 ± 1.3					
Thin limbs^b^	Inner and outer medulla	AQP‐1	1.1 ± 0.3		**+**	**+**		
Thick ascending tubule	Outer medulla		7.3 ± 1.4		**+**	**+**		**+**
Papillary duct^c^	Papilla		8.6 ± 1.2		**+**	**+**	**+**	
Urothelium (top layer)		CYTK8‐18	17.5 ± 3.5		**+**	**+**	**+**	

a Cell height: mean ± standard deviation

b In thin limbs, the cell height was measured in regions without visible nuclei

c In the papillary ducts, the cell height was measured at the broadest site of the cell.

### Glomerulus and Bowman's capsule

**Figure 4:**
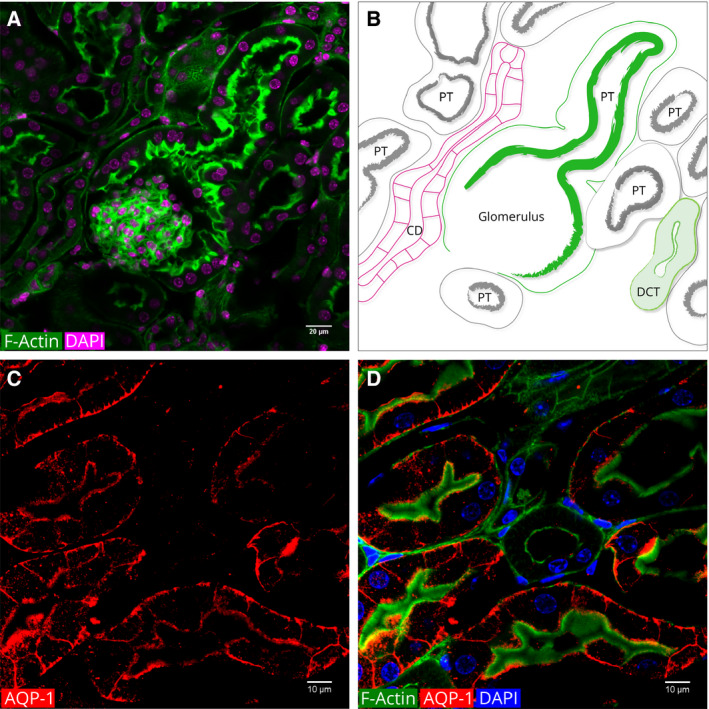
Actin cytoskeleton patterns in the glomeruli, Bowman's capsule, and PTs. (A) A merged image of DAPI‐stained nuclei and phalloidin fluorescence of actin filaments. (B) Schematic diagram of A. The color of the DAPI was switched from blue to magenta to enhance contrast. The intensely stained glomerulus (Fig. 3) is surrounded by Bowman's capsule. At the renal pole, the Bowman's capsule opens into the PT. The luminal side of both the Bowman's capsule and the PT has thick bundles of actin filaments. These filaments run along the brush border membranes. CD: collecting duct. DCT: distal convoluted tubule. (C) Immunofluorescence of AQP‐1 in PTs. (D) Merged image of phalloidin and AQP‐1 and DAPI IF. PTs are characterized by intense fluorescence of thick actin filaments on the luminal border. Note that the round smaller tubule in the center of the image shows no AQP‐1 IF. This tubule has the characteristics of a DT.

A Bowman's capsule envelopes each glomerulus. The parietal epithelial cells that cover the inner surface of the Bowman's capsule showed a thick line of actin at the luminal border and a thin line at the basal border (Fig. 4). The luminal surfaces of the parietal cells are covered with microvilli 40. Thus, the thick line of actin fluorescence must be due to the actin bundles of the microvilli of the parietal cells.

**Figure 5:**
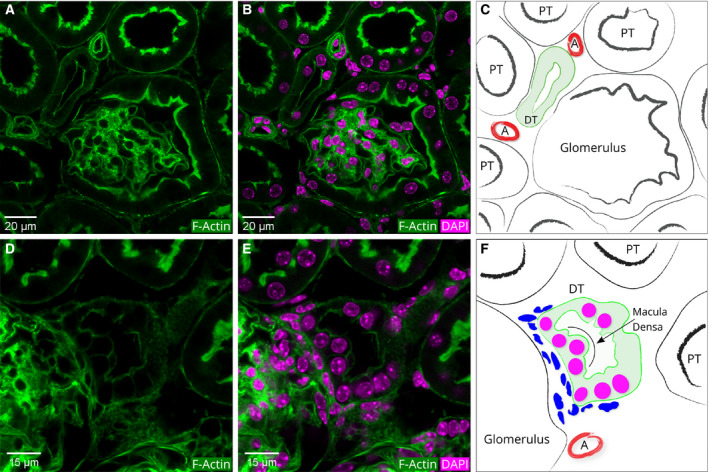
A magnified view of the vascular pole of a glomerulus and a macula densa region. (A) Phalloidin fluorescence of actin filaments. (B) Merged image of DAPI and phalloidin fluorescence. (C) Schematic diagram of A. The afferent and efferent arterioles at the vascular pole appear brightly stained due to their smooth muscle lining (marked with an ‘A’ in the schematic diagram). The luminal side of the PT is lined with a brush border membrane rich in actin filaments. Consequently, the luminal border of the PTs shows intense phalloidin fluorescence. In contrast, the DT located at the vascular pole has only a thin bundle of actin filaments lining the luminal side. (D, E, and F) Macula densa region of a nephron. (D) Phalloidin fluorescence of actin filaments. (E) Merged image of DAPI and phalloidin fluorescence. (F) Schematic diagram of D. Note that the cells in the macula densa are densely packed and have prominent round nuclei.

The Bowman's capsule has two poles. At the vascular pole, the afferent arterioles enter the glomerulus. The blood that is filtered exits via the efferent arterioles that are juxtaposed to the afferent arterioles. We could visualize these arterioles with their brightly stained borders, as these arterioles have an actin filament‐rich smooth muscle lining 41 (their locations are marked as ‘A’ in Fig. 5C).

### Proximal tubules

The filtrate that flows into the Bowman's space drains into the opening of the PT (Fig. 4). This site is called the urinary pole. Relative to the vascular pole, the urinary pole is on the opposite side of the Bowman's capsule.

To identify the PTs, we used anti‐AQP‐1 that is specifically expressed in PTs 22. Immunofluorescence of AQP‐1 was observed on both luminal and basal borders of the PTs but neither in DTs nor in CDs (Fig. 4C,D).

In Fig. 4, it can be seen that the actin fluorescence of the PT luminal border appears as a continuation of the thick actin fluorescence of the Bowman's capsule. The apical surface of the PTs is covered with microvilli that make up the so‐called brush border membranes. In mouse PT sections, we measured the height of the microvilli as 2.55 ± 0.28 μm.

**Figure 6:**
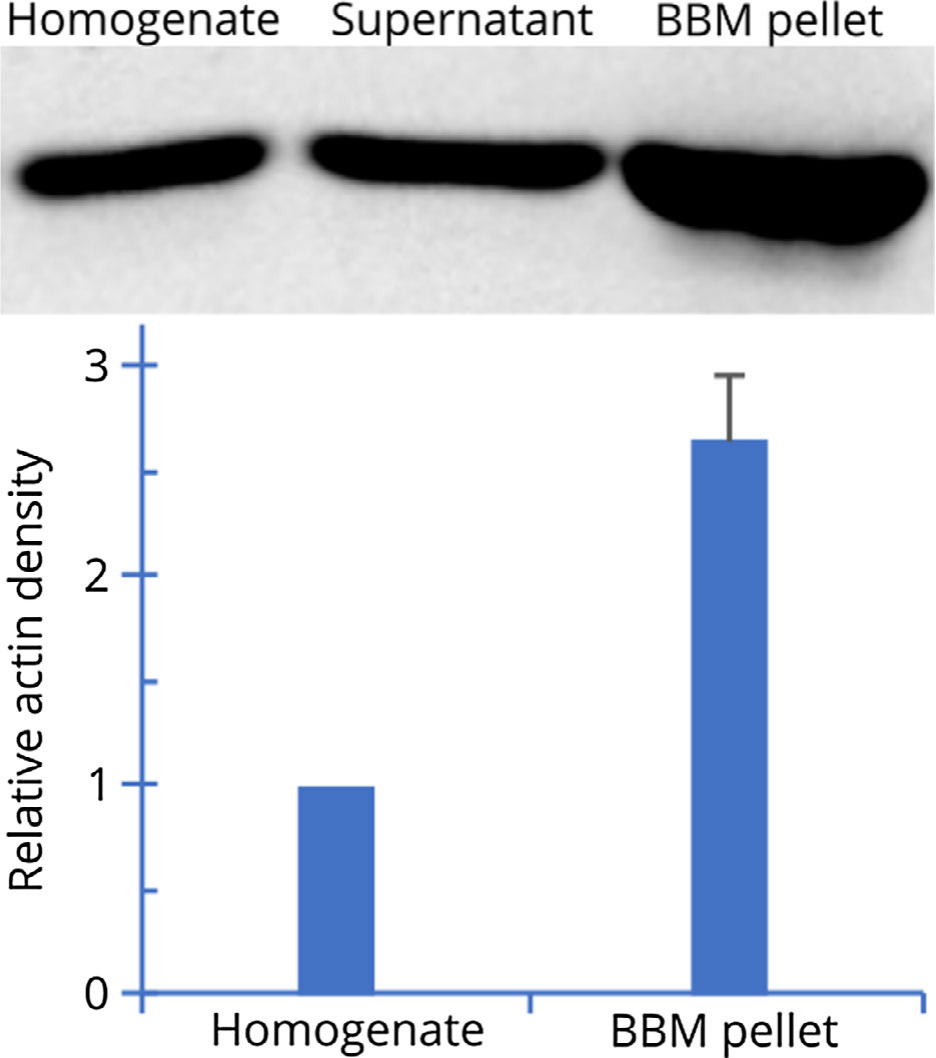
Quantitation of actin in brush border membrane (BBM) fraction by WB analysis. Kidney brush border membrane fraction was prepared as described in Materials and methods. Three lanes of the gel were loaded with 20 µg of total protein from total kidney homogenate, final supernatant, and the BBM fraction, respectively. After electrophoresis, the proteins were transferred to a nitrocellulose membrane and then reacted with anti‐β‐actin as described in Materials and methods. The major actin band was ~ 42 kDa in all samples. Densitometric analysis of the WB of four independent experiments showed that in the BBM fraction, average actin intensity was 2.65 (SEM: 0.31) times higher than that in the whole kidney homogenate.

Each microvillus has a backbone of an actin bundle that is composed of 19 actin filaments 42. To examine the actin content, we isolated brush border membranes as described in Materials and methods. Densitometric quantitation of the WB of the total kidney homogenate and subcellular fractions using anti‐β‐actin antibodies showed that the brush border membrane fraction has ~ 2.7 times more actin relative to the whole mouse kidney homogenate (Fig. 6).

**Figure 7:**
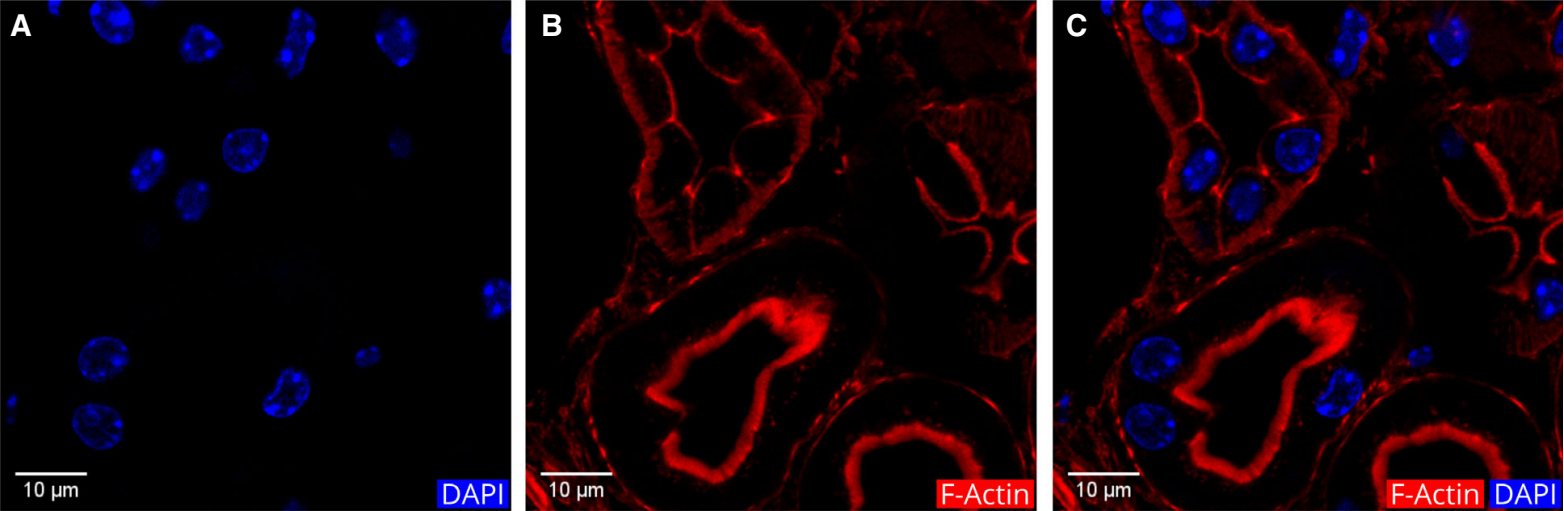
Control staining of a cortical region with DyLight 554 phalloidin. (A) DAPI staining of the cell nuclei, (B) actin filament staining, (C) merged image of A and B. The two tubules with an intense luminal border staining are PTs (at the lower half of the image).

Based on these results, we identified the tubules that have a thick apical actin fluorescence as PTs. To confirm the reliability of the fluorescence, we reacted a mouse kidney section with a different phalloidin conjugated with DyLight 554. This phalloidin with red fluorescence yielded immunofluorescence (IF) images similar to that of the previous results (Fig. 7).

### Distal tubules (DT)

**Figure 8:**
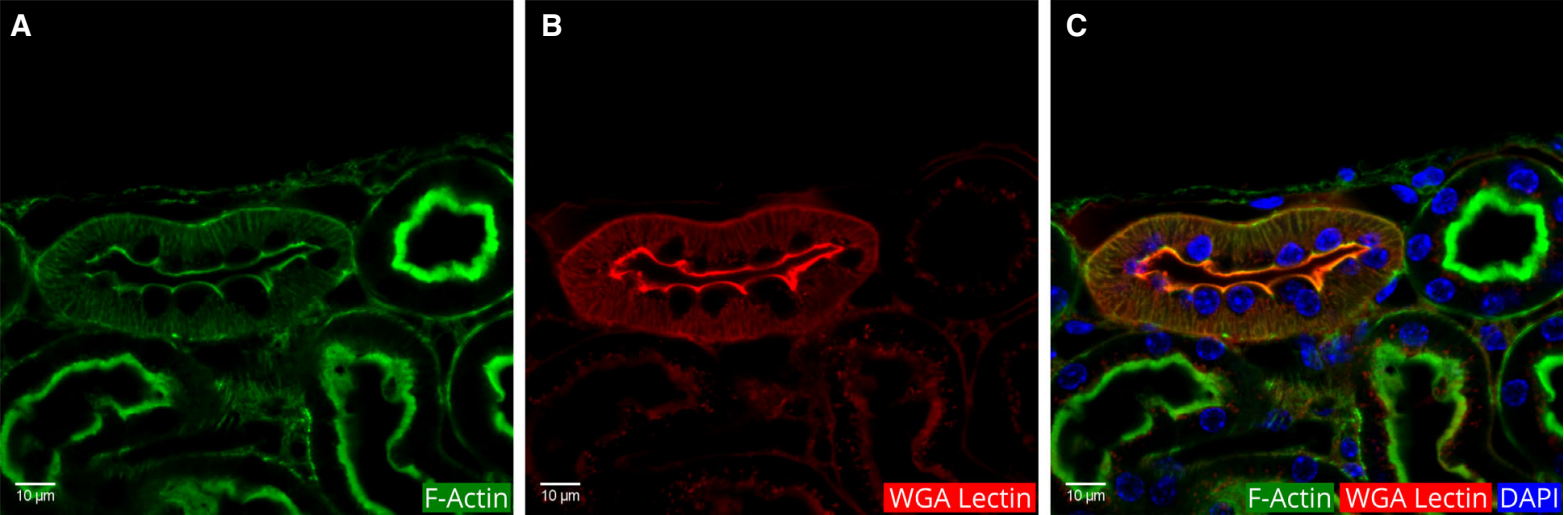
Identification of a DT in a cortical region by its actin cytoskeleton pattern and WGA lectin fluorescence. (A) Phalloidin fluorescence of actin filaments. (B) WGA lectin fluorescence. (C) Merged image of DAPI, actin, and lectin fluorescence.

The second type of actin cytoskeleton pattern in the cortex appeared as a thin line on both the apical and basal borders (Fig. 8). We identified these as DT segments because of their locations and additional features noted below. Aguirre *et al*.^43^ had shown that in kidney sections, WGA lectin binds most strongly to the apical border DTs. Based on these results, we employed fluorescent WGA lectin as a marker for DTs. Indeed, the tubules we identified as DT showed intense fluorescence on the apical border (Fig. 8). In PTs neighboring the DT, much weaker lectin fluorescence was observed in vesicles close to the lumen (Fig. 8).

### Juxtaglomerular apparatus

As shown in Fig. 1, a small segment of the distal straight tubule is juxtaposed to the Bowman's capsule at the vascular pole. Such a typical DT section appears in Fig. 5 in between the two arterioles marked with ‘A’ (Fig. 5C). Note that both the apical and the basal borders of this tubule have a thin line of actin cytoskeleton distinctly different from those of the PTs (Fig. 5). This is the site of the juxtaglomerular apparatus (JGA) that is responsible for renin secretion. The JGA is composed of three types of epithelial cells:

- Macula densa: A segment of the distal straight tubule (thick ascending tubule) wherein the cells bulge into the lumen near the vascular pole of the glomerulus as shown in Fig. 5F. Macula densa cells have large round nuclei and are densely packed (purple colored nuclei in Fig. 5F).
- Extraglomerular mesangial cells and granular cells: These cells have elongated nuclei and are located between the macula densa and the Bowman's capsule (blue colored nuclei in Fig. 5F). We could not distinguish between these two types of cells based on their actin cytoskeleton.

The region we have identified displays all the features of the JGA (Fig. 5).

After the JGA, the distal straight tubule is followed by the distal convoluted tubule (DCT) that continues to have a thin actin cytoskeleton on both the apical and basal borders as shown in Fig. 8. An additional characteristic of the DCT is very thin intracytoplasmic (IC) stripes of actin filaments (Fig. 8).

### Collecting ducts in the cortex

**Figure 9:**
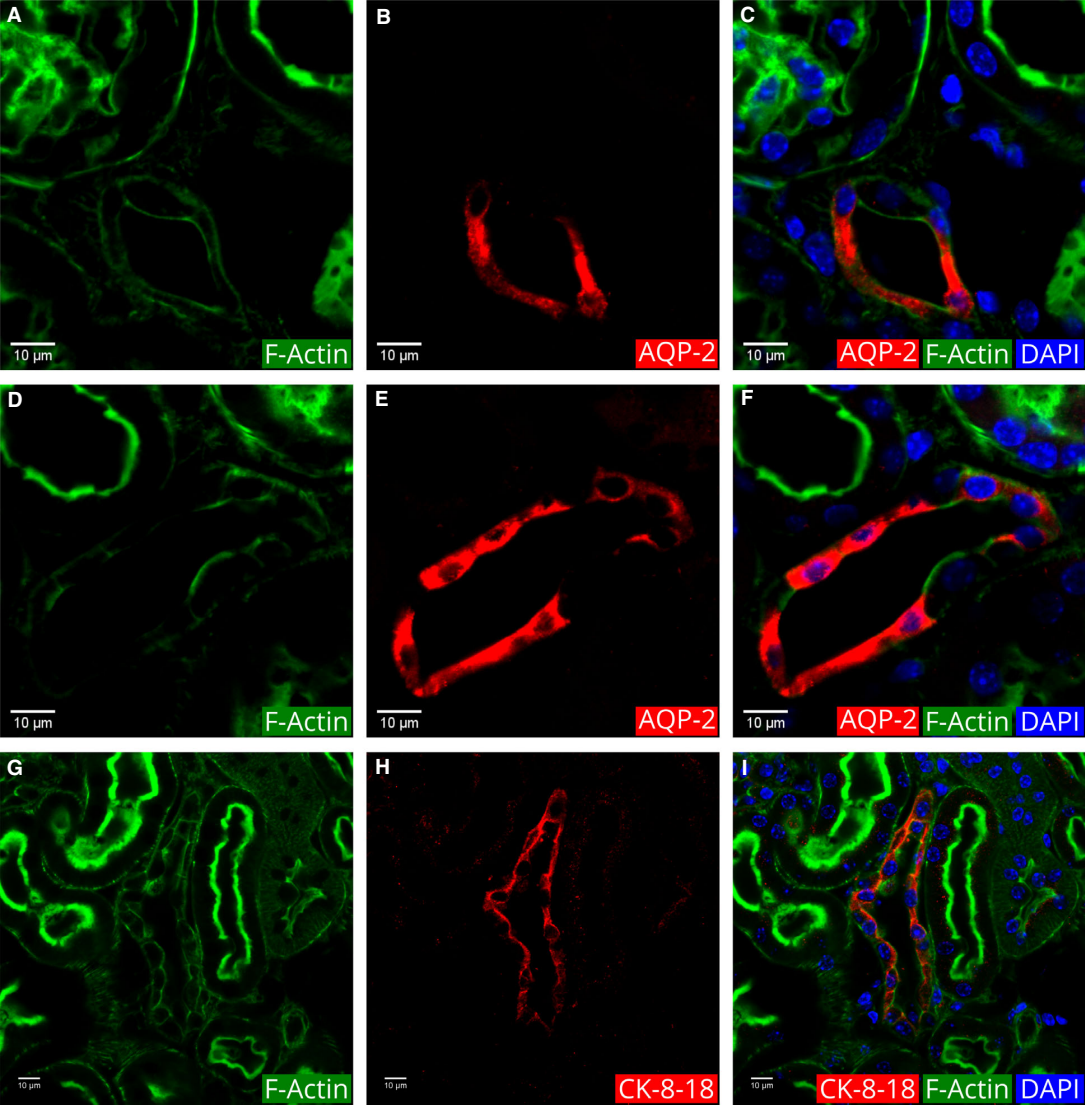
Identification of the connecting tubules and the CDs in the cortex using anti‐AQP‐2 and anti‐cytokeratin‐8‐18. (A, D, and G) Actin filament fluorescence. (B and E) Anti‐AQP‐2 IF. (H) Anti‐CK‐8‐18 IF. (C, F, and I) Merged image of the first two images with DAPI. The top row includes images from a region with a connecting tubule adjacent to a glomerulus. The middle row includes images from a region with a CD. The images in all rows include PTs with strong apical border actin (green) fluorescence.

In addition to the PT and DT epithelia, there is a third type of tubule epithelia found in the cortical region with actin filaments on both the apical and basal borders, and also on the lateral border between cells (Fig. 9A,D,G).

Previous studies showed that the principal cells in the CDs specifically express AQP‐2 44, 45. Based on these findings, we reacted the kidney sections with anti‐AQP‐2 and saw that specifically these tubules with actin filaments on the lateral borders also express AQP‐2 (Fig. 9B,E). Thus, we identified these tubules as the CDs in the cortex (Table 1).

In the CD epithelia, some cells were not stained by anti‐AQP‐2 antibodies (Fig. 9). The distribution of actin filaments in these two types of epithelial cells appeared different. The cells that were not stained by anti‐AQP‐2 antibodies are the intercalating cells. The distribution of actin filaments on the apical border of intercalating cells appeared relatively thicker compared to that of the principal cells (Fig. 9F). The intermediate filament proteins, keratin‐8 and keratin 18, are strongly expressed in CD epithelia 46. The reaction of anti‐cytokeratin 8‐18 antibodies with our sections showed the strongest fluorescence in the tubules that we identified as the CDs in the cortex (Fig. 9H).

### Medulla

**Figure 10:**
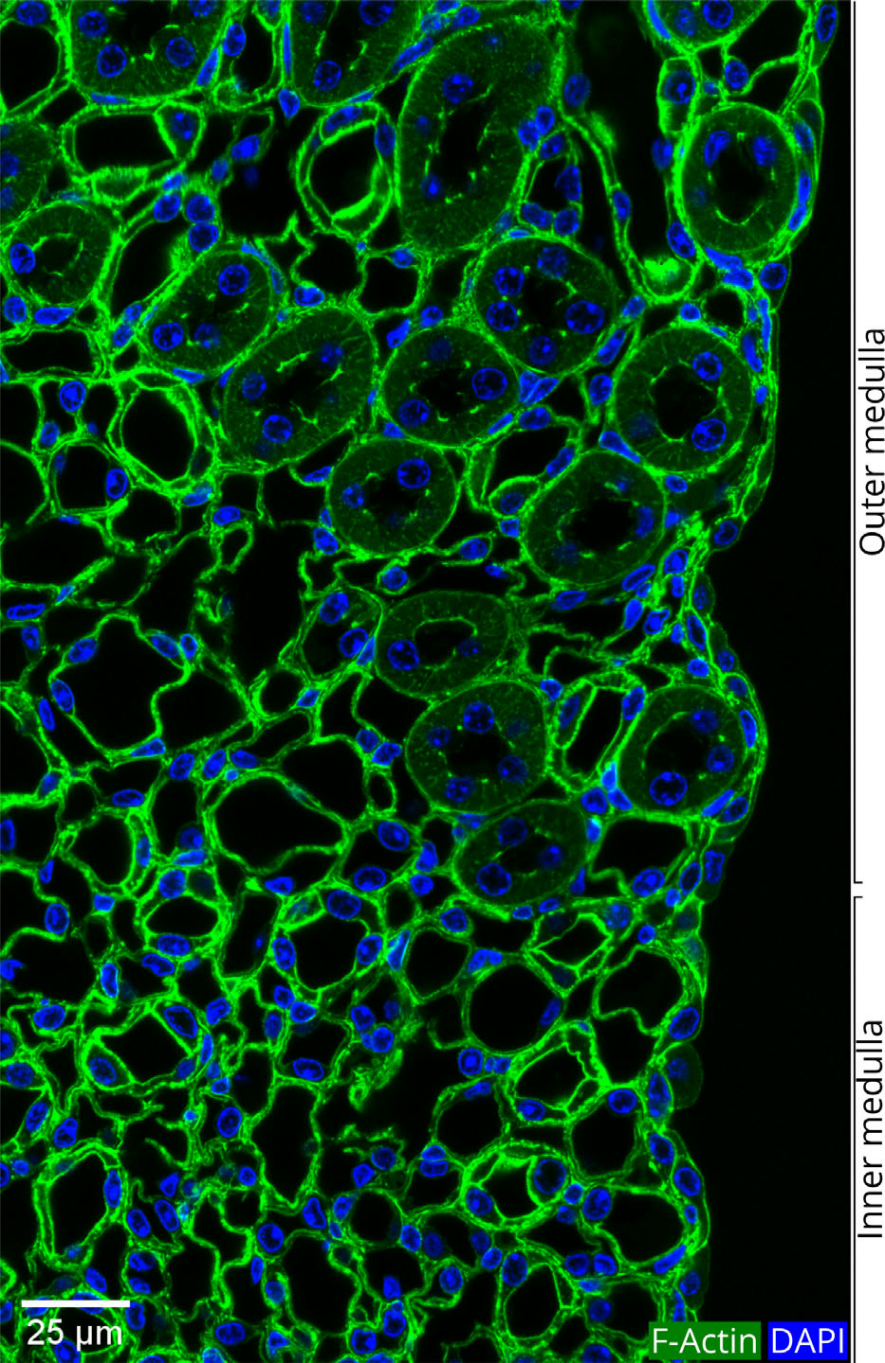
The transition zone between the outer and inner medullary regions. Both the outer and inner medulla contain tubules that form the descending thin limb and the ascending thick limb of Henle's loop and the CDs. The thin tubules can be easily distinguished from the thick ascending limbs as the cell height in the thin tubules is very short (~ 1 μm), and the cell height in the thick tubules is much longer (~ 7 μm) (Table 1). Outer medulla is characterized by the presence of thick ascending limbs. A single‐layered epithelial covering is visible at the right edge of this section.

**Figure 11:**
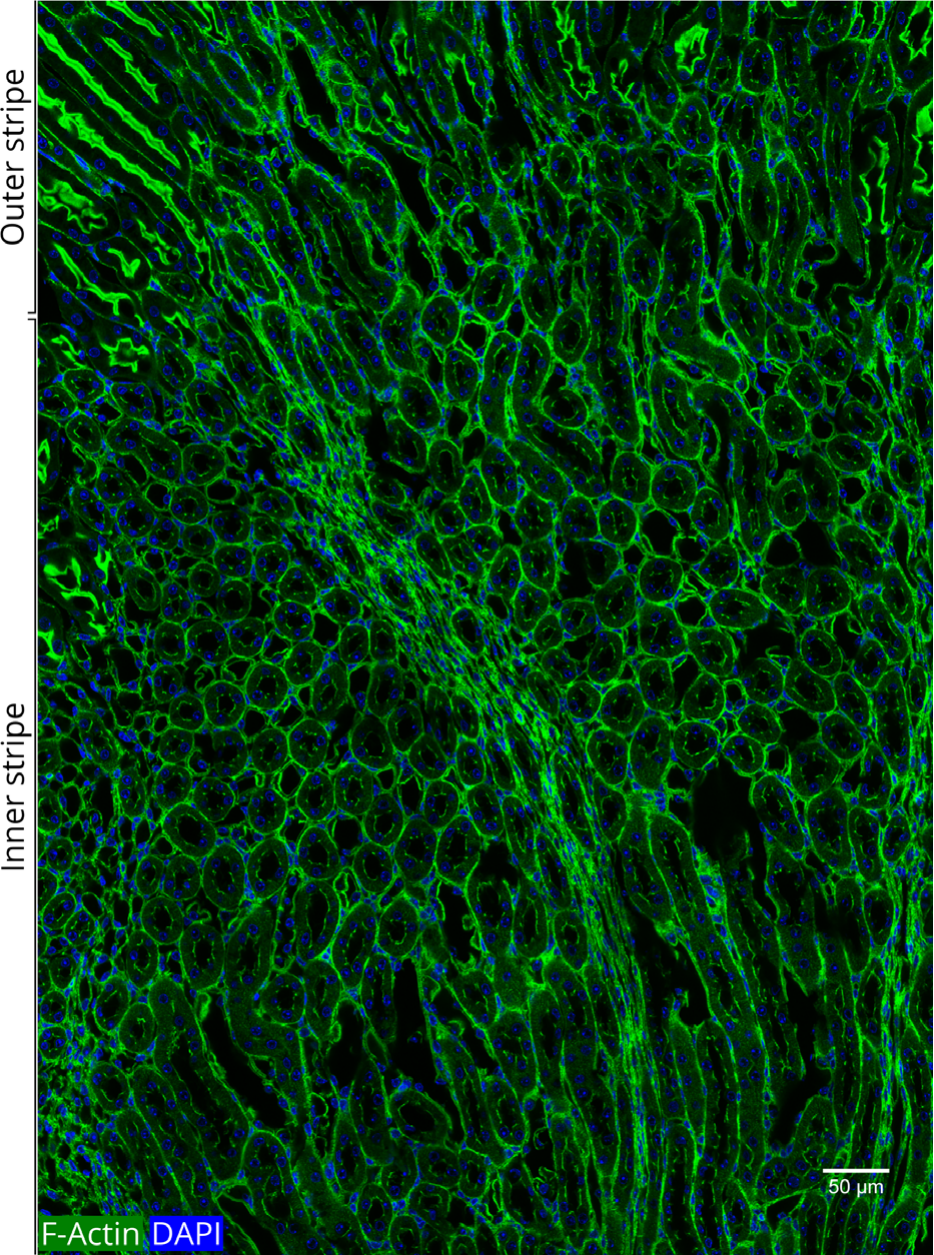
Distinctive morphology of the outer medullary zones visualized by actin filaments. This image shows the transition zone between the outer stripe and the inner stripe of the outer medulla (see Figs. 1 and 2). The outer stripe is characterized by elongated tubules with a thick bundle of apical actin filaments. In contrast, the tubules in the inner stripe are round with a thin patchy lining of actin filaments. The medullary rays passing through the inner stripe are characterized by the thin and elongated shape of the tubules and the cell nuclei.

The overall pattern of the actin cytoskeletal architecture and the morphological features of the epithelia in the medulla are very different from those of the cortex (Fig. 2). We found three distinct patterns of actin cytoskeletal structure in the epithelial cells of the medullary region (Figs 10 and 11).

- Thin elongated epithelial cells with actin cytoskeleton on both the apical and basal borders that may appear as a single thread,
- Thin and patchy apical, and thin and continuous basal actin filaments, and
- Thin actin filaments on the apical, basal, and lateral borders.

As detailed below, we associated these three patterns of epithelia with (a) thin limb of Henle's loop, (b) thick ascending limb of Henle (distal straight tubule), and (c) CDs, respectively. As can be seen in the nephron diagram (Fig. 1), thin and thick limbs, and CDs are located in parallel. Thus, in a cross section of the kidney in the region of the outer medulla, all three of these tubules were visible (Fig. 11). In the inner medulla, only the thin limbs and the CDs were seen (Fig. 10).

Both the outer and the inner medulla (Fig. 2), and papilla have an outer covering of a single‐layered epithelium (Fig. 10) 47.

### The thin limb of Henle's loop

In the nephron structure, the PT transforms into the thin limb at the border between the outer and inner stripes of the outer medulla (Fig. 1). The thin limbs were distinguished from the thick limbs by their very short cell height as compared to the thick ascending limbs (1.1 vs. 7.3 μm; Table 1). In a general view of the outer and inner medulla, these differences were easily visualized (Fig. 10).

**Figure 12:**
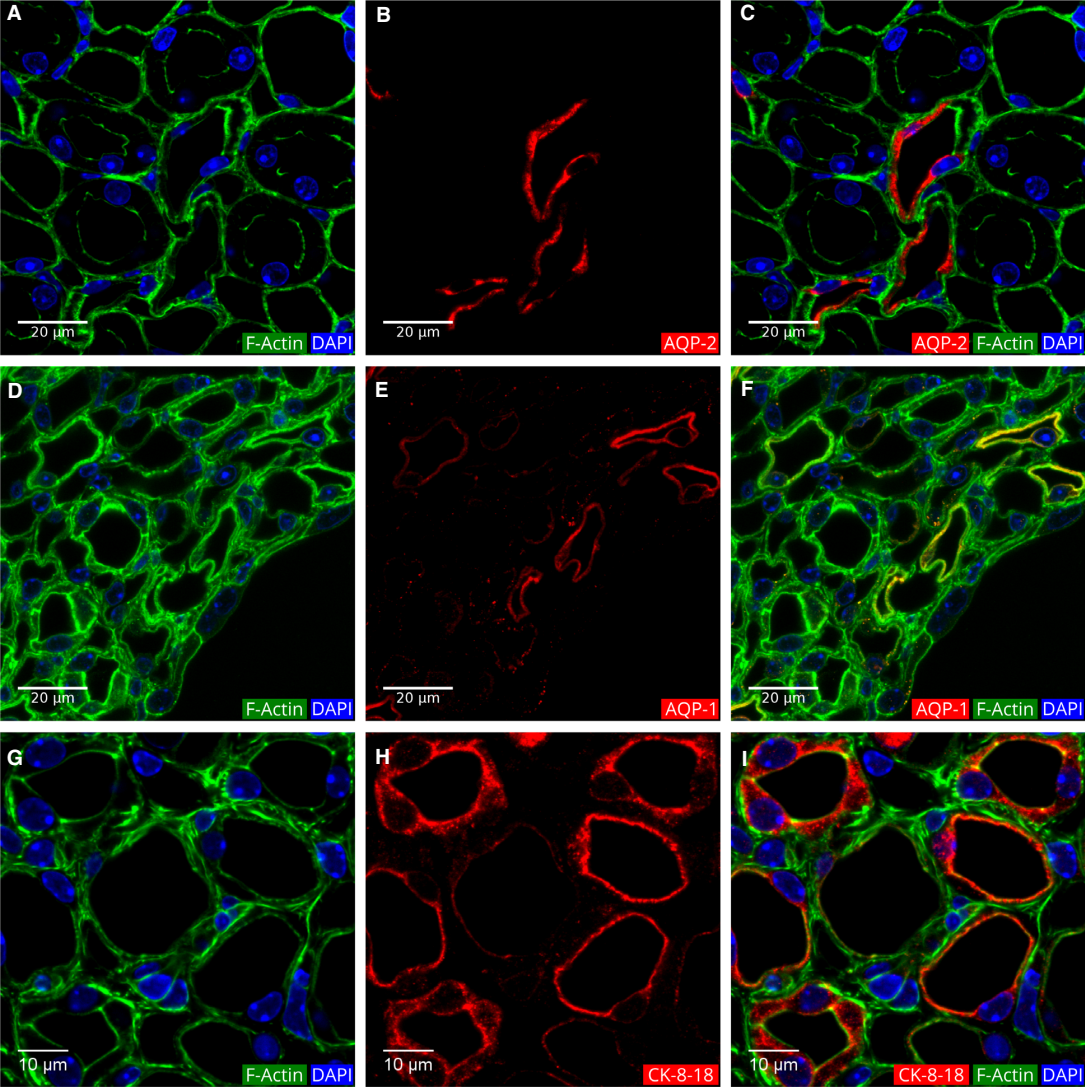
Identification of the CDs and thin limbs in the medulla. (A, D, and G) Actin filament and DAPI fluorescence. (B, E, and H) Anti‐AQP‐2, anti‐AQP‐1, and anti‐CK‐8‐18 IF, respectively. (C, F, and I) Merged image of the first two images. Note that the CDs have a thin lining of actin filaments at apical, basal, and intercellular borders. Thin bundles of actin filaments line both the apical and basal membranes of the thin limbs. The single‐layered epithelial lining covering the inner medullary region is also clearly distinguishable in D. In the medulla, CK‐8‐18 is present in the CDs and in some thin loop segments.

**Figure 13:**
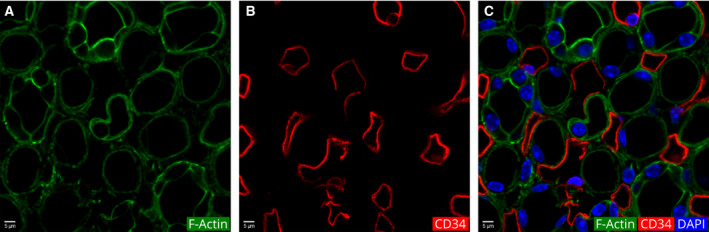
Visualization of endothelial cells in the blood vascular system of the inner medulla using anti‐CD34 antibodies. (A) Actin fluorescence. (B) CD34 IF. (C) Merged image of A and B with DAPI.

In addition to the structural differences, the thin limbs were also characterized by their expression of AQP‐1 (Fig. 12D–F). A subset of the thin limbs showed intense IF of cytokeratins 8 and 18, while the rest of the thin limbs showed weak IF (Fig. 12G–I). In addition, the CD34 IF showed that the ascending and descending vasa recta in the medulla are present in spaces where there is no visible actin fluorescence (Fig. 13).

### Thick ascending limbs of Henle's loop

The name thick ascending limb reflects the fact that the epithelial cells of this segment are tall (Table 1), resulting in thick tubules (Fig. 10). A major distinguishing feature of the cells of the thick ascending limbs is that their apical border shows only a patchy lining of actin filaments (Table 1, Fig. 10).

As seen in Figs 10 and 11, the cells of the thick ascending limb and DT epithelia include a fine IC network of actin filaments. The PT sections are devoid of such filaments (Fig 7).

### Collecting ducts in the medulla

As described above for the CDs in the cortex, the CD segments in the medulla were also identified by their anti‐AQP‐2 and keratin 8‐18 IF (Fig. 12). These tubules were also made up of principal and intercalating cells with only the principal cells staining for anti‐AQP‐2 antibodies. The apical lining of the actin filaments of the intercalating cells appears slightly thicker than the apical actin filament lining of the principal cells.

### Identification of the nephron segments by actin cytoskeleton patterns

**Figure 14:**
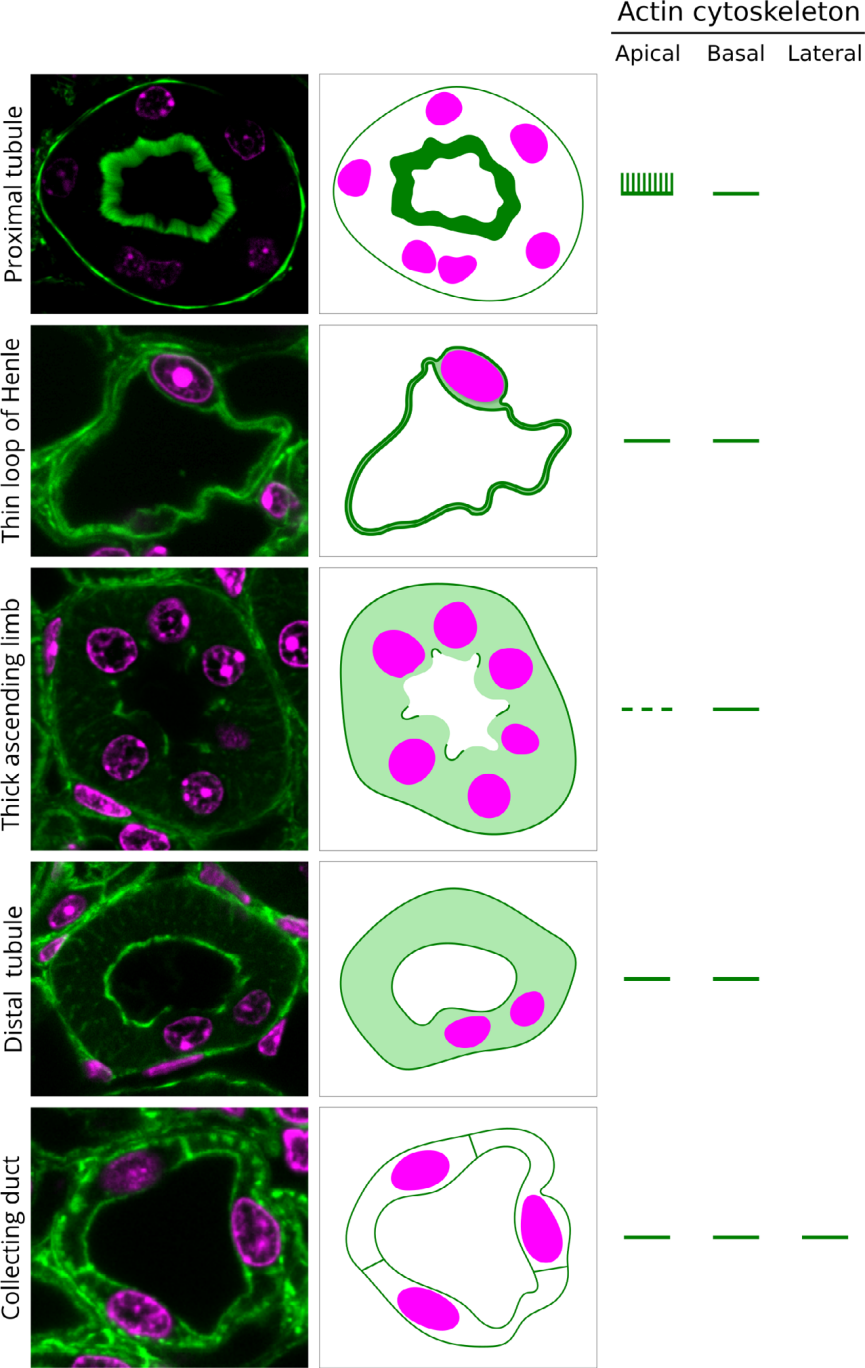
Examples of the epithelial cell types in the kidney. The left panel shows examples of the epithelial section from the following tubules: (1) PT, (2) thin limb of Henle, (3) thick ascending limb of the Henle, (4) DCT, and (5) CD. The schematic diagrams in the midpanel and the lines in the right‐most panel show the features of the actin filaments on the apical, basal, and lateral borders of the epithelial cells. See Table 1 for morphometric data about all five types of cells.

To summarize the results described above, in Fig. 14 we present examples of actin cytoskeleton fluorescence for five distinct tubular epithelia: (a) PT, (b) thin limb of Henle's loop, (c) thick ascending limb of Henle's loop, (d) DCT, and (e) CD. On the right side of Fig. 14, we indicated the type of filaments present on the apical, basal, and lateral borders of the tubule segment. This figure together with the information in Table 1, summarizing cell characteristics, can be used to identify renal tubule segments.

### The renal papilla

**Figure 15:**
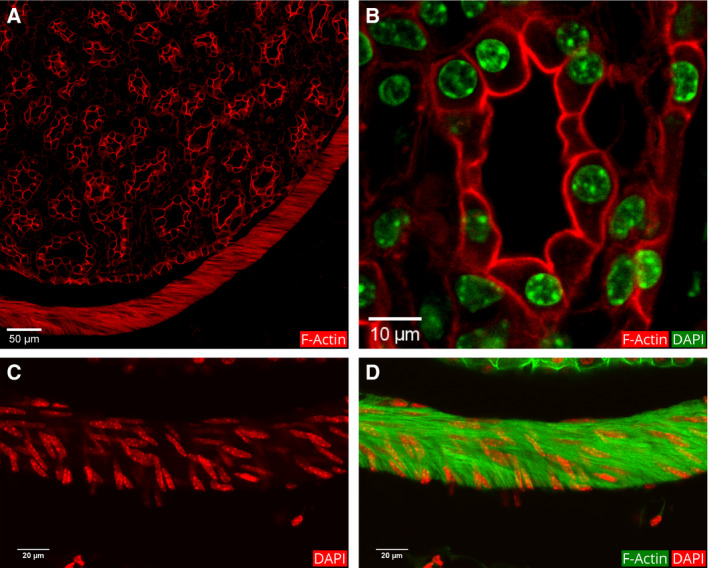
Distribution of actin filaments in the papillary region. (A) This image shows only the phalloidin fluorescence that marks the actin filaments. The fluorescence color was switched from green to red to enhance contrast. Thin actin filaments clearly demarcate the borders of the cells in the papillary ducts. The red strip of actin bundles surrounding the papilla is a smooth muscle lining. (B) This magnified image shows merged actin and DAPI staining of nuclei in one papillary duct. To enhance the contrast, the DAPI color was changed to green, and phalloidin color to red. Thin bundles of actin filaments line the apical, basal, and lateral intercellular borders of the cells of papillary ducts. The nuclei appear round and smooth. (C and D) Magnified view of the smooth muscle lining. Note the elongated shape of the nuclei in C and the dense localization of the actin filaments in the merged image (D) showing both the DAPI and actin fluorescence.

The renal nephrons terminate at the CDs that merge, forming larger tubules called papillary ducts (also known as ducts of Bellini) 48. These ducts form the papilla that is the final route for urine that drains into the pelvic space and from there into the ureter. To visualize the papilla, we took a section from a midsection of a mouse kidney. Phalloidin fluorescence of this section, including papilla, showed a tubular organization that is distinctly different from that of other renal regions. Actin cytoskeletons in the papillary ducts showed a uniform pattern with filaments at the apical, basal, and lateral borders (Fig. 15B). The surface of the papilla is covered by a single‐layered epithelium (Fig. 15).

A striking feature of the section is an intensely stained band of actin bundles that form a sac‐like structure around the papilla (Fig. 15D). The nuclei of the cells that make up this sac have an elongated structure (Fig. 15C). This is probably the smooth muscle lining of the ureteropelvic border.

### Urothelium

**Figure 16:**
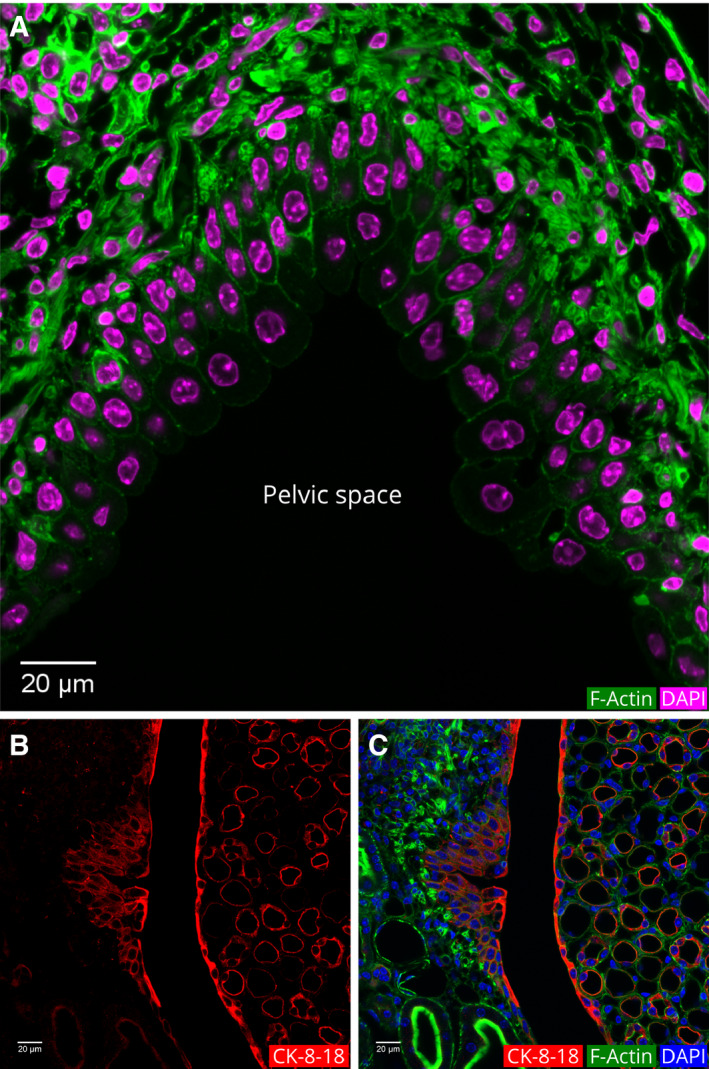
Cellular morphology and localization of actin filaments in the pelvic region. (A) Actin filaments and DAPI fluorescence in the urothelial cells and smooth muscles. The urothelium is multilayered and columnar at the pelvis. Actin filaments line the borders of these cells. (B) Cytokeratin‐8‐18 IF in the urothelial cells and the inner medullary region. (C) Merged image of B with actin filament and DAPI fluorescence.

The papillary ducts drain into the renal pelvis. The pelvis is lined by a specific type of multilayered epithelium named urothelium (Fig. 16). Compared to the tubular epithelial cells, the apical layer of the urothelium has the biggest cells with a mean cell height of 17.5 ± 3.5 µm (Table 1). Some of the cells of the urothelium are binucleated, and a few other cells appear in different stages of nuclear separation as previously described 49.

The superficial layer of the urothelium (also known as umbrella cells) has actin filaments on all cell borders. The intensity of the actin filament fluorescence on the basal and lateral borders is higher than that on the apical border. A unique feature of the urothelium actin cytoskeleton is the appearance of an intense actin fluorescence on the apical end of the lateral border. This bright spot may be indicative of the tight junctions at this point (Fig. 16).

The superficial cell lining of the inner medulla and the urothelia showed intense IF of the anti‐cytokeratin 8‐18 antibodies, distinguishing it from the rest of the renal cellular architecture (Fig. 16).

## Discussion

In this study, for the first time, we generated a map of the actin cytoskeleton architecture in sections of a whole mouse kidney by high‐resolution confocal microscopy of phalloidin fluorescence. In these maps, the cortex, the outer and inner stripes of the outer medulla, and the inner medulla could be demarcated just by the appearance of the actin filaments (Fig. 2). By a higher resolution structural analysis of these maps, we could distinguish and identify all the major nephron components and segments, including the glomeruli, Bowman's capsule, PTs, thin and thick limbs of Henle's loop, DCTs, and CDs and papillary ducts. The structural features we used to identify the nephron segments included the following: (a) the luminal and basal thickness of actin filaments; (b) localization of tubules and cells relative to glomeruli; (c) shapes of the nuclei; and (d) IC actin filaments. Figure 14 presents examples of the epithelial cells of the nephron segments and their actin cytoskeleton patterns. Table 1 summarizes the cellular morphology in these segments. The nephron segments could be identified using the features noted. For further verification of the structural identifications, we also used IF of AQP‐1 and AQP‐2 isoforms, keratin 8‐18, WGA lectin, and CD34 43, 44, 46, 50, 51.

Some features of the actin cytoskeleton we observed by phalloidin fluorescence, especially in the glomerulus and the brush border membrane, have been previously reported 52, 53, 54, 55. However, none of these studies reported comprehensive criteria for the identification of major nephron segments. Some studies identified nephron tubular segments using agglutinin and multiple immunohistochemical stainings in renal sections 56, 57. The approach we described here is much simpler, provides much higher resolution, and does not include the background histochemical staining of a whole section.

### Structure and functional significance of the actin cytoskeleton

In epithelial cells, the apical membrane is supported by a membrane skeleton that is composed of a network of actin filaments cross‐linked by spectrins and other actin‐binding proteins 30, 58. As can be seen in our summary figure (Fig. 14), all the epithelial cells of the nephron segments have both an apical and a basal actin cytoskeleton. There are clear differences between the apical and basal actin cytoskeletons of these segments (Fig. 14).

The thick and fuzzy luminal surface fluorescence in the PTs reflects the undular surface of the brush border that is composed of densely packed microvilli (Figs 4 and 14). The microvilli in the rat kidney proximal convoluted tubule are uniform in size, and their length has been measured as 2.7 μm 33. In our images of the mouse PT, the average height of the microvilli appears as ~ 2.6 μm. The core of each microvillus is estimated to include 19 actin filaments arranged in a hexagonal structure wherein the filaments are cross‐linked by fimbrin and villin 42. These microvilli greatly increase the membrane surface area of the PT to facilitate the processes of absorption of electrolytes and solutes 5.

Another essential function of the microvilli is acting as sensors for the axial flow of glomerular filtrate in the lumen of the PT. The hydrostatic pressure in the afferent arteriole is about twofold higher than the pressure in the efferent arteriole 59. This difference contributes to the intraluminal hydrostatic pressure that pushes the flow glomerular filtrate through the PT. It has been shown that an increase in the rate of perfusion rate via mouse PT increases the inner diameter of the tubule as microvilli bend 59. The actin bundle backbone of the microvillus is also linked to the membrane by myosin1A: calmodulin linkers 30, 42.

The actin cytoskeleton that lines along the plasma membrane has been shown to have essential roles in epithelial cell function including the processes of endocytosis and exocytosis, and direct and indirect (via actin‐binding proteins) interactions with membrane‐bound transporters, channels, and AQP isoforms 30, 60.

The actin cytoskeleton of the epithelial cells of the thick ascending limbs and the DCTs differ from other tubules by the presence of what appears as IC actin filaments (Figs 10 and 14). The cells in the thick ascending limb and DT epithelia have amplified basolateral membranes with interdigitated processes that are filled with mitochondria 2, 61. In a tubule cross section, these processes are aligned with the appearance of a palisade embedded in the basolateral membrane 61. Thus, what appears as IC actin filaments represents the actin filaments that line the basolateral membrane of the interdigitated processes.

The most distinct actin cytoskeleton difference between the CD epithelia and the preceding segments of the nephron is the consistent appearance of actin filaments along the lateral borders of the epithelia at cell–cell interaction sites (Figs 9 and 14). Compared to the PT, the DT and the CDs represent a tight epithelium with low ion permeability 7, 62. While in the PT, a significant percentage of the fluid absorption proceeds via paracellular pathways, in the CDs, because of the tight junctions between cells, the paracellular route is virtually blocked 62. On the cytoplasmic side, the molecules that form the intercellular linkage are anchored directly or indirectly to actin filaments via actin‐binding proteins 30, 63. Thus, the actin filaments we observe specifically on the lateral side of CD epithelia most likely represent the actin cytoskeleton to which the focal adhesion is anchored beneath the tight junction.

In summary, in this study, we have generated a map of the actin cytoskeleton of a whole mouse kidney, and we have documented distinct differences in the actin cytoskeleton patterns of renal nephron epithelia along the entire nephron from the glomerulus to the CDs. The features of the cytoskeletal differences we documented are consistent with the current knowledge of the structure and function of nephron tubule segments. The simple approach we employed here using phalloidin fluorescence of actin filaments can be used in identifying and classifying epithelia in other mammalian tissues as well to identify various cell types in them.

Renal diseases and the transition from AKI to chronic kidney disease are generally followed by serum biomarkers such as creatinine 37. To understand the pathophysiology of these diseases, many animal models have been developed 64, 65. The approach presented in this study could be useful for the identification of the structural damages in kidney tubules in various renal diseases and animal models.

## Materials and methods

### Tissue preparation and cryosectioning

All tissues were obtained from adult male ICR mice weighing between 40 and 60 g (aged 6–12 weeks). The animals were housed at 25 °C with a 12‐h dark/light cycle. All animals had *ad‐libitum* access to standard food and water. The study protocol was approved by the Institutional Animal Ethics Committee of Ariel University (permit 32_12733_b019), according to the Ministry of Health guidelines.

The kidneys for cryosectioning were obtained from mice after perfusion. The mice were anesthetized with an intraperitoneal injection of 100 mg·kg^−1^ ketamine and 5 mg·kg^−1^ xylazine. Cardiac perfusion was performed using PBS, pH 7.4, followed by 4% paraformaldehyde w/v (BDH Limited, Poole, UK) dissolved in PBS. The kidneys were removed, cut into 2‐ to 4‐mm blocks, and left in 4% paraformaldehyde in PBS overnight at 4 °C. The tissues were then transferred into 30% sucrose w/v dissolved in PBS at 4 °C for 2–3 days until all the tissue blocks settled at the bottom of the tube.

The tissues were then embedded in a cryomedium OCT compound (Tissue‐Tek, Sakura, the Netherlands) and frozen at −20 °C. A Slee MEV Floor Standing ECO Cryostat (SLEE Medical GmbH, Mainz, Germany) was used for cryosectioning at a chamber temperature of −25 °C and object temperature of −20 °C. The tissue sections (15–30 μm thick) were collected in PBS containing 0.1% sodium azide in a 24‐well plate.

### Fluorescent labels, antibodies, and immunofluorescence

After cryosectioning, the tissue sections were placed in 4% paraformaldehyde in PBS for 20 min. The fixative was suctioned out, and sections were washed with PBS twice for 5 min. The tissue sections were then permeabilized with 0.1% v/v Tween‐20 (Sigma‐Aldrich, St. Louis, MO, USA) in PBS for 10 min and washed again with PBS for 5 min. All tissue sections were then blocked with 2% w/v BSA in PBS (incubation buffer) for 20 min. All the experiments were performed at room temperature and under dim light.

The fluorescent labels and antibodies we used are listed in Table 1. For actin filament staining, the sections were incubated with 1 : 20 dilution of phalloidin in incubation buffer for 60 min. After the incubation, the sections were washed three times with PBS, 5 min each. For the WGA lectin reaction, the lectin was added together with phalloidin.

Marker antibodies were added to the sections at a dilution indicated in Table 2 and incubated in incubation buffer for 60 min. The sections were then washed in PBS six times for 5 min each. Immediately after washing, the tissue sections were incubated with Alexa Fluor 555 secondary antibody (1 : 500) in incubation buffer for 60 min, and then washed with PBS six times 5 min each. This was followed by actin filament staining as described above.

**Table 2:** Fluorescent labels and antibodies used in the experiments.

Fluorescent labels/antibodies	Host, company, and Catalog No.	Application–dilution
Phalloidin, CF488A conjugate	Biotium	F‐1 : 20
DyLight 554 Phalloidin	Cell Signaling Technology, #13054	F‐1 : 20
Anti‐β‐actin	Rabbit polyclonal, Abcam, #AB227387	WB‐1 : 2000
Anti‐AQP‐1	Rabbit, Abcam, #AB15080	IF‐1 : 100
Anti‐AQP‐2	Rabbit, Alomone Labs, #AQP‐002	IF‐1 : 100
Anti‐CD34	Rabbit, Abcam, #AB81289	IF‐1 : 100
Anti‐cytokeratin 8‐18	Guinea pig, Abcam #AB194130	IF‐1 : 50
Alexa Fluor 555 IgG (H + L)	Goat anti‐rabbit, Life Technologies, #A21428	IF‐1 : 500
Alexa Fluor 555 IgG	Goat anti‐guinea pig, Abcam, #AB150186	IF‐1 : 200
Peroxidase conjugate IgG (H + L)	Goat anti‐rabbit, Jackson ImmunoResearch, #125510	WB‐1 : 1000
WGA lectin CF558 conjugate	Biotium, #29076‐1	F‐1 : 300

The nuclei were stained with DAPI (4′6‐diamidino‐2‐phenylindole) at a 1 : 100 dilution in incubation buffer for 2 min as the last step of the process. The samples were then washed with PBS for 5 min at least two times and then mounted onto X‐tra adhesive slides (Leica Biosystems, Peterborough, UK) using the anti‐fade reagent n‐propyl gallate (Sigma‐Aldrich) 0.5% (w/v) in 100 mm phosphate‐buffered (pH 7.2) glycerol (90%).

All experiments were performed at least three times with independent samples.

### Confocal microscopy

Fluorescent‐labeled kidney sections were visualized using an LSM 700 confocal microscope (Carl Zeiss, Germany). All images were collected at 405 nm excitation for DAPI, 488 nm excitation for CF488A, and 555 nm excitation for Alexa Fluor 555. Fluorescent and bright‐field illumination modes were also used during the image acquisition process. Tile‐scan images were collected using 15‐μm‐thick sections and LCI Plan‐Apochromat 25×/0.8 oil objective lens, with 25–50% overlap. The number of tiles was set depending on the surface area to be scanned. During the process of tile scanning, the stage is moved automatically based on the scanning speed. Tile‐scan images were stitched using Carl Zeiss (Jena, Germany) zen 2012 software at a correlation threshold of 0.40. The images were viewed by imagej software (https://imagej.nih.gov/ij/) and optimized for brightness and contrast.

### Morphometric measurements

The lengths of the epithelial cells and the microvilli were measured using imagej. We measured cell height only for tubules that appeared to be cut at a right angle to the tubule axis. We confirmed our visual evaluation by 3D z‐stack images of the tubular sections. For each tubule segment, five images from independent experiments were analyzed. Fifteen measurements were taken from each tubule segment. The lengths of the microvilli were measured in five different images with ten measurements from each. For the papillary ducts, measurements were taken on 25 cells from five different tubules. The lengths of the luminal cells of the urothelia were measured in 14 cells. The results are presented as mean ± standard deviation.

### Isolation of brush border membranes

Proximal tubule brush border membranes were isolated as described 66. Two entire mouse kidneys from two different animals were extracted, cut into pieces, and transferred into two Eppendorf tubes filled with 0.8 mL freshly prepared homogenization buffer (300 mm D‐mannitol, 5 mm EGTA, 12 mm Tris, and 0.5 mm PMSF, adjusted to pH 7.1 at 4 °C). The kidneys were then homogenized using DI 18 Basic (IKA, Staufen, Germany) for 2 min. The homogenate was immediately mixed with ice‐cold 1.12 mL Millipore water containing 12 mm MgCl_2_ and left on ice for 15 min. The mix was centrifuged at 1500 ***g*** at 4 °C. The supernatant was decanted into a new Ultra Microtube (Thermo Scientific, Waltham, MA, USA) and centrifuged at 25 000 ***g*** using a F50L‐24×1.5 rotor (Thermo Scientific) in a Sorvall wX + Ultra Series Centrifuge (Thermo Scientific) set at 4 °C for 30 min. The pellet was resuspended in Tris buffer (50 mm Tris, 0.5 mm PMSF, and 1 mm EGTA, pH 7.4 at 4 °C), and the membranes were collected by centrifugation at 25 000 ***g*** for 30 min. The pellet was again resuspended in 1 mL ice‐cold Tris buffer. Samples were collected at each step and stored at −80 °C immediately.

### Western blot analysis

The protein concentration was determined by Bio‐Rad Protein Assay (Bio‐Rad, Hercules, CA, USA) based on the Bradford method using BSA as standard. The protein samples were dissolved in sample buffer (50 mm Tris/HCl, pH 6.8, 2% SDS, 0.2% bromophenol blue, 10% glycerol, and 5% β‐mercaptoethanol). The samples were incubated at 98 °C for 5 min and then separated by electrophoresis on an 8.5% SDS/PAGE gel, and transferred onto a nitrocellulose membrane (Bio‐Rad). The membrane was incubated with the blocking solution (1% fat milk) for 90 min at room temperature. The membrane was then incubated with the anti‐β‐actin antibody (Table 2) with a 1 : 2000 dilution in the blocking solution for 12–14 h at 4 °C with gentle shaking. After incubation, the membrane was washed six times with PBST (0.1% Tween‐20 in PBS, 10 min each). After washing, the membrane was incubated with HRP‐conjugated goat anti‐mouse IgG (1 : 2000) in blocking solution for 90 min at room temperature with gentle shaking. The membrane was washed six times with PBST for 10 min each. The peroxidase‐labeled membrane was developed using luminol as described (Enuka *et al*. 23).

## Conflict of interest

The authors declare no conflict of interest.

## Author contributions

GKK carried out the experiments. Both GKK and IH participated in data collection and analysis and manuscript writing.
